# Seroprevalence of SARS-CoV-2 (COVID-19) exposure in pet cats and dogs in Minnesota, USA

**DOI:** 10.1080/21505594.2021.1936433

**Published:** 2021-06-14

**Authors:** Mythili Dileepan, Da Di, Qinfeng Huang, Shamim Ahmed, Daniel Heinrich, Hinh Ly, Yuying Liang

**Affiliations:** a Department of Veterinary & Biomedical Sciences, College of Veterinary Medicine, University of Minnesota, Twin Cities, USA; b Department of Veterinary Clinical Sciences, College of Veterinary Medicine, University of Minnesota, Twin Cities, USA

**Keywords:** COVID-19, SARS-CoV-2, cat, dog, seroprevalence, Elisa, neutralization antibodies, feline coronaviruses, zoonoses

## Abstract

The COVID-19 pandemic caused by the coronavirus SARS-CoV-2 is continuing to spread globally. SARS-CoV-2 infections of feline and canine species have also been reported. However, it is not entirely clear to what extent natural SARS-CoV-2 infection of pet dogs and cats is in households. We have developed enzyme-linked immunosorbent assays (ELISAs) using recombinant SARS-CoV-2 nucleocapsid (N) protein and the receptor-binding-domain (RBD) of the spike protein, and the SARS-CoV-2 spike-pseudotyped vesicular stomatitis virus (VSV)-based neutralization assay to screen serum samples of 239 pet cats and 510 pet dogs in Minnesota in the early phase of the COVID-19 pandemic from mid-April to early June 2020 for evidence of SARS-CoV-2 exposures. A cutoff value was used to identify the seropositive samples in each experiment. The average seroprevalence of N- and RBD-specific antibodies in pet cats were 8% and 3%, respectively. Among nineteen (19) N-seropositive cat sera, fifteen (15) exhibited neutralizing activity and seven (7) were also RBD-seropositive. The N-based ELISA is also specific and does not cross react with antigens of common feline coronaviruses. In contrast, SARS-CoV-2 antibodies were detected at a very low percentage in pet dogs (~ 1%) and were limited to IgG antibodies against SARS-CoV-2 N protein with no neutralizing activities. Our results demonstrate that SARS-CoV-2 seropositive rates are higher in pet cats than in pet dogs in MN early in the pandemic and that SARS-CoV-2 N-specific IgG antibodies can detect SARS-CoV-2 infections in companion animals with higher levels of specificity and sensitivity than RBD-specific IgG antibodies in ELISA-based assays.

## Introduction

The new severe acute respiratory syndrome coronavirus 2 (SARS-CoV-2) emerged in late 2019 in Wuhan, China, and is causing the COVID-19 pandemic [1,2] that has profound health, social, and economic impacts on a global scale. As of May 2021, there are >150 million confirmed COVID-19 cases worldwide and >3 million deaths associated with SARS-CoV-2 infection, including >32 million cases and >575 thousand deaths in the US alone. No effective antiviral compounds are currently available. Monoclonal antibodies (mAbs) can be used to treat mild to moderate COVID-19 patients but not severe disease [3]. Several vaccines, including mRNA-based vaccines by Pfizer/BioNTech and Moderna as well as adeno viral vector-based vaccine by Johnson & Johnson, have been approved by FDA for emergency use authorization (EUA) [4]. However, the emergence and wide spread of new SARS-CoV-2 variants that can reduce the effectiveness of the current vaccines pose significant threat to combating the pandemic.

SARS-CoV-2 has 80% amino acid sequence identity to SARS-CoV that caused the 2003 SARS outbreak with ~10% fatality rate [2]. Both are members of the genus Betacoronavirus that also include Middle East respiratory syndrome virus (MERS-CoV), which emerged in 2012 and caused severe respiratory illness with ~ 36% fatality rate [5]. All three highly pathogenic CoVs have been reported to originate in bat populations and are believed to transmit to humans through intermediate animal hosts, such as palm civets for SARS-CoV and camels for MERS-CoV, but the potential intermediate host(s) for SARS-CoV-2 remain unknown [6]. SARS-CoV-2, like other coronaviruses, is an enveloped RNA virus with a single-stranded RNA genome of ~ 31 kb that encodes 16 non-structural proteins (nsp1-nsp16), 4 structural proteins (spike S, envelope E, membrane M, and nucleocapsid (N)), and several accessory proteins [7]. The spike (S) protein of SARS-CoV-2 protruding from the viral envelope membrane is responsible for viral entry by binding to human angiotensin-converting enzyme 2 (hACE2), the same receptor used by SARS-CoV, through the receptor-binding domain (RBD) on the spike protein and is responsible for mediating the virus-cell membrane fusion [8–12]. SARS-CoV-2 can also infect many animal species [13]. Ferrets, Syrian golden hamsters, domestic cats, cynomolgus macaques, and raccoon dogs have been shown to be highly permissive to SARS-CoV-2 after experimental inoculation and can shed and transmit the virus to co-housed (sentinel) animals [14–21]. Most of the infected animals show no clinical signs and develop none or mild signs of respiratory inflammation, except for Syrian hamsters that can develop severe pathological lung lesions [15,21]. Dogs can also be experimentally infected with SARS-CoV-2 but do not shed virus, whereas pigs, chickens, and ducks are not at all susceptible to experimental SARS-CoV-2 infections [14,19,22]. Natural infections by SARS-CoV-2 have been reported in pet dogs, cats, zoo tigers, and lions that show only mild respiratory signs [23–25]. In contrast, SARS-CoV-2 has caused widespread and lethal infections in farmed mink in the EU and in the USA [26,27]. Outbreaks of SARS-CoV-2 infections in both mink and humans on mink farms suggest that the virus can potentially easily cross the natural species barrier between mink and men, raising the concern for zoonosis and reverse zoonosis of SARS-CoV-2 transmission [28]. The recent isolation of a SARS-CoV-2 mink-associated variant strain with decreased sensitivity to neutralizing antibodies and its infection of more than 750 people so far have led to the culling of 17 million farmed mink in Denmark [28–30]. These zoonotic new variants have also raised a real concern over its impact on the efficacy of the COVD-19 vaccines either in use or currently under various stages of development and testing [31,32].

There are estimated 76 million pet dogs and 96 million pet cats living in approximately 70% of the U.S. households [24 and references therein]. Therefore, these companion animals have very close contacts with humans in close quarters. As companion animals are the potential sources and sentinels of a wide range of infectious diseases, determination of their susceptibility to and prevalence for natural SARS-CoV-2 infections has significant impacts for both animal and human health [33–35]. We therefore undertook a serological survey of 239 cat sera and 510 dog sera collected in the Veterinary Medical Center (VMC) at the University of Minnesota, Twin Cities in the early period of the COVID-19 epidemic in Minnesota (MN) from mid-April to mid-June of 2020. The IgG antibodies in these pet sera against the SARS-CoV-2 N and spike RBD were measured by indirect ELISAs developed in our laboratory. The levels of neutralizing antibodies (nAbs) in the pet sera against SARS-CoV-2 were also quantified by using a SARS-CoV-2 spike-pseudotyped VSV-based neutralization assay. We have detected higher seroprevalence in pet cats than in pet dogs in MN early in the COVID-19 pandemic. Moreover, SARS-CoV-2 N-based ELISA detected more seropositive samples, which were corroborated by neutralization assay, than RBD-based ELISA.

## Materials and Methods

### Animal serum samples

De-identified serum samples were obtained from discarded patient serum samples, which were collected for routine diagnostics of pet cats and dogs for illness, wellness or chronic disease monitoring at the Veterinary Medical Center (VMC) of the University of Minnesota, Twin Cities. Serum samples of 510 pet dogs and 239 pet cats in total were collected between mid-April and mid-June of 2020, heat inactivated at 56°C for 30 min, and stored at −20°C until use. It was reported that sera inactivated by heating the serum samples at 56°C for 30 min could minimize the risk of virus transmission/contamination of laboratory staff, but did not impair the positive detection rate of SARS-CoV-2 ELISA [36]. No identifiable information about the pets and their owners were made available to the researchers aside from the animal species. The owners of these pets have signed informed consents to allow discarded biological samples (i.e., sera) from their pets to be used in this study.

### Production of recombinant nucleocapsid (N) protein and the receptor-binding domain (RBD) of SARS-CoV-2

**Figure 1.**
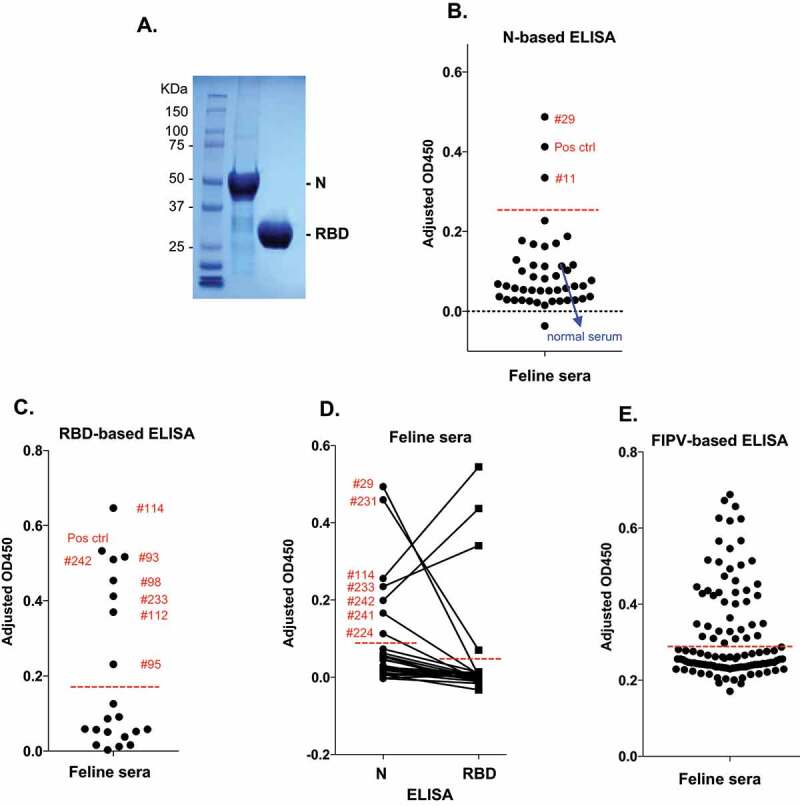
**Serological tests of pet cat sera by ELISA**. (a) Purified recombinant SARS-CoV-2 N and RBD proteins shown in SDS-PAGE gel after Coomassie blue staining. (b) A representative SARS-CoV-2 N IgG ELISA with pet cat sera. Normal cat serum purchased from a commercial source, the positive control (SARS-CoV N-specific mAb 1C7C7), and two seropositive samples (#29 and #11) are shown. (c) Pet cat sera tested by RBD IgG ELISA. The positive control (mAb 1C7C7), and seropositive samples are shown. (d) A batch of pet cat sera were tested with both N and RBD IgG ELISA. None of the RBD-positive sera are N-negative. The ID# of N seropositive samples are shown. (e) Evaluation of pet cat sera with IgG ELISA against feline infectious peritonitis virus (FIPV) antigens. Each serum was tested pairwise in uncoated and coated wells in technical duplicates. The adjusted OD_450_ value was calculated by subtracting OD_450_ value of uncoated well from that of the coated well. The cutoff OD_450_ value was calculated as described in Materials and Methods and shown as a red dash line

The full-length SARS-CoV-2 N gene was synthesized by Twist Biosciences (San Francisco, CA) and cloned into the bacterial expression vector pRSF-Duet1 with an N-terminal His tag and a C-terminal Strep tag. BL21(DE3) bacterial cells transformed with the plasmid were grown at 37°C and induced overnight with 1 mM IPTG. The cell pellet was collected by centrifugation at 5,000 g in F15-8x50cy fixed angle rotor for 20 min and resuspended in the denaturing buffer (20 mM Na_2_HPO_4_, pH 7.4, 500 mM NaCl, and 6 M urea). Cells were lysed by sonication followed by centrifugation at 25,000 g for 60 min. The supernatants were filtered and applied onto Histrap HP column (GE healthcare) with AKTApure chromatography system. The column was washed with the denaturing buffer, followed by the renaturing buffer (20 mM Na_2_HPO_4_, pH 7.4, 500 mM NaCl) and the HisTrap wash buffer (20 mM Na_2_HPO_4_, pH 7.4, 500 mM NaCl, 50 mM imidazole) before being eluted with 10x column volume of the HisTrap elution buffer (20 mM Na_2_HPO_4_, pH 7.4, 500 mM NaCl, and 500 mM imidazole). The fractions containing the recombinant N protein were pooled and dialyzed against PBS buffer overnight. The purified SARS-CoV-2 N protein is free of associated RNA as determined by the OD260/280 ratio and quantified by the Bradford assay. SARS-CoV-2 RBD was expressed from mammalian cells as described previously [37] and was a kind gift of Y. Wan and F. Li, University of Minnesota, Twin Cities. Both recombinant proteins were of high quality as shown in the SDS-PAGE gel that was stained by Coomassie blue dye (Figure 1a).

### SARS-CoV-2 N- and RBD-based indirect enzyme-linked immunosorbent assays (ELISAs)

Maxisorp plates (96 wells) (Thermo Fisher Scientific, Cat# 437,111) were either un-coated or coated with 100 ng of the purified N or RBD protein in bicarbonate buffer overnight at 4°C. Wells were blocked with 5% nonfat milk in PBS with 0.05% Tween20 (PBST) for 1 h at 37°C. Heat-inactivated sera were diluted to 1:50 with 1% nonfat milk in PBST, added equally to both uncoated and coated wells, and incubated for 1 h at 37°C. Monoclonal antibodies against RBD (CR3022, recombinant human mAb obtained from M. Jenkins, University of Minnesota, Twin cities) or against SARS-CoV N protein (1C7C7L mouse mAb obtained from L. Martinez-Sobrido, Texas Biomedical Research Institute) were included as positive controls. Normal feline (Cat# 102,643; Jackson Immunoresearch Laboratories Inc.) and canine sera (Cat# 004–000-001; Jackson Immunoresearch Laboratories Inc.) were included as negative controls. Following the wash with PBST, HRP-conjugated goat anti-cat IgG polyclonal antibody (Cat # RL602-1302; Rockland Immunochemical) or goat anti-canine IgG polyclonal antibody (Cat# A18763; Thermo Fisher Scientific) was added at a 1:1000 dilution and incubated for 45 min at 37°C. Following the addition of substrate ABTS (Cat# A9941; Millipore Sigma) and stop solution (3 N HCl), the absorbance at 450 nm (OD_450_) was measured using the Synergy2 multiplate reader (BioTek). Normalized OD_450_ of each sample was calculated by subtracting OD_450_ of un-coated well from OD_450_ of coated well. The cutoff value was set as the mean plus three standard deviations of the values obtained from the negative samples. Each serum sample was screened in at least two independent experiments.

### ELISA to detect feline infectious peritonitis coronavirus (FIPV)

An indirect ELISA was set up to detect FIPV antibodies in the cat sera. A total of 116 samples were tested, including all 19 serum samples positive for N-ELISA and additional 97 randomly selected serum samples to evaluate the seroprevalence of FIPV in these pet cats. The native FIPV antigens (catalog # MBS568874, MyBioSource) were used to coat the plate at 200 ng/well in bicarbonate coating buffer at 4°C overnight. The plate was blocked with 5% nonfat milk in PBST for 1 h at 37°C. Heat-inactivated cat serum samples were diluted at 1:50 in PBST with 1% nonfat milk and added into both FIPV-coated and un-coated wells. Goat anti-FIPV polyclonal antibody (catalog # MBS560925, MyBioSource) was included as a positive control. The HRP-conjugated goat anti-cat (Cat # RL602-1302; Rockland Immunochemical) and mouse anti-goat (cat # sc-2354, Santa Cruz) secondary antibodies were used at 1:1000 dilution in PBST with 1% nonfat milk. After 10 min – 15 min of substrate incubation at 37°C, the stop solution (3 N HCl) was added. Normalized OD_450_ was obtained and analyzed as described above.

### SARS-CoV-2 spike-pseudotyped vesicular stomatitis virus (VSV)-based neutralization assay

A replication-defective VSV with SARS-CoV-2 spike protein was used to measure the neutralizing antibody titers in the cat’s and dog’s sera following the procedure as previously described [38]. To generate SARS-CoV-2 spike-pseudotyped recombinant VSV, human kidney epithelial (293 T) cells on 10 cm^2^ plate were transfected with the plasmid pSARS-CoV-2Δ19 expressing the SARS-CoV-2 spike protein (a kind gift of T. Hatziioannou and P. Bieniasz, Rockefeller University), and 24 h later infected with the seed virus rVSVΔG/Fluc that contains the VSV-G and expresses the firefly luciferase gene (a kind gift of S. Paessler, University of Texas at the Medical Branch), in the presence of I1-Hybridoma (ATCC #CRL-2700) supernatants that contain anti-VSV-G monoclonal antibody. The supernatants were collected 24 h after infection and ultracentrifuged at 27,000 rpm in SW28 rotor for 2 h. The rVSVΔG/Fluc-CoV2-S viral particle pellets were resuspended in PBS, aliquoted, and stored at −80°C. For the neutralization assay, the rVSVΔG/Fluc-CoV2-S pseudotyped virus was incubated with serum samples in serial dilutions for 30 min and applied to 293 T/ACE2(B) stable cells (a kind gift of T. Hatziioannou and P. Bieniasz, Rockefeller University) on 96-well plates, in the presence of anti-VSV-G monoclonal antibody. At 16 h post-infection, cells were lysed and the FLuc activity was quantified using the Firefly Luciferase Assay System following the manufacturer’s protocol (Promega). The neutralization titer is determined as the serum dilution that reduces the FLuc activity by 50%. To assess whether the pseudotyped viral entry was mediated by SARS-CoV-2 Spike in an hACE2-dependent manner, 293 T/ACE2(B) cells were first incubated with increasing concentrations (0, 2.5, 5, 10, 20, and 40 ug/ml) of recombinant hACE2 proteins or bovine serum albumin (BSA) as a negative-control protein, before the addition of the rVSVΔG/Fluc-CoV2-S pseudotyped virus. The FLuc activity was quantified as described above.

## Results

### Screening feline serum samples by SARS-CoV-2 N-based ELISA

The nucleocapsid (N) protein encapsidates viral genomic RNA and is one of the most abundant SARS-CoV-2 proteins, whereas spike (S) protein is responsible for mediating virus entry into infected cells [7]. As N and S proteins are known main targets of antibody responses in human COVID-19 patients [39,40], current serological assays are mostly based on the N and S or the immunogenic epitopes of S [41]. Using recombinant full-length SARS-CoV-2 N protein purified from bacteria that is free of any associated RNAs, we have developed an N-based ELISA to detect SARS-CoV-2 IgG antibodies in serum samples of pet cats, which were collected in separate batches at the Veterinary Medical Center of the University of Minnesota, Twin Cities between mid-April to mid-June 2020 (Table 1). Each serum sample was applied onto un-coated and N-coated wells of the 96-well plate to conduct the ELISA as described in the Materials and Method and to obtain OD_450_ measurements. The adjusted OD_450_ values for all serum samples were calculated using the simple formula: OD_450_ (N coated-well) – OD_450_ (un-coated-well). As shown in Figure 1b, most representative samples, including a normal feline serum as a negative control, have adjusted OD_450_ values that are below 0.25, while three of the samples including the feline serum samples #11 and #29 and a positive control sample that contains an anti-N mAb showed significantly higher OD_450_ values. Based on these results, the OD_450_ cutoff value used to distinguish positive samples from negative samples was set as the mean OD_450_ values plus three standard deviations of those of the negative samples and shown as a red dashed line (Figure 1b), which classified feline serum samples #11 and #29 as positive for anti-N antibodies. Out of 239 cat sera screened, 19 samples were deemed to be positive for anti-N antibodies in this N-based ELISA, which yielded an overall seroprevalence of 7.9%. These feline positive serum samples include 2 samples obtained from April 6 to 27 April 2020; 14 samples from May 8 to 2 June 2020; and 3 samples from June 5 to 12 June 2020 (Table 1). There is a noticeable increase of seroprevalence for N-specific IgG antibodies in pet cats since mid-May, 2020 (0–5% seroprevalence before and 11–12% seroprevalence after mid-May) that appears to correspond to an increase in the numbers of COVID-19 in human populations in MN [42]. However, due to the lack of information on the pet owners, it is not clear whether there is a direct correlation between infected pet cats and their owners.

**Table 1.** Feline serum samples screened by SARS-CoV-2 N- and RBD-based ELISAs

feline serum ID#	Collection date	N ELISA (#)	N sero-positive (#)	N sero-positive rate* (CI)	RBD sero-positive (#)	RBD sero-positive rate* (CI)
**1–43**	4/16/20 – 4/27/20	43	2	4.7% (0.6, 15.8)	0	0% (0.0, 8.2)
**44–89**	4/28/20 – 5/8/20	46	0	0.0% (0.0, 7.7)	0	0.0% (0.0, 7.7)
**90–218**	5/8/20 – 6/2/20	125	14	11.2% (6.3, 18.1)	5	4.0% (1.3, 9.1)
**219–243**	6/5/20 – 6/12/20	25	3	12.0% (2.5, 31.2)	2	8.0% (1.0, 26.0)
**Total**		239	19	7.9% (4.9, 12.1)	7	2.9% (1.2, 5.9)

* Proportions are reported with 95% confidence interval (CI) computed using the Clopper-Pearson method.

### Screening feline serum samples by SARS-CoV-2 RBD-based ELISA

To validate the SARS-CoV-2 N-specific IgG antibody responses in cat sera, we developed an RBD-based IgG ELISA in order to detect RBD-specific antibodies by using recombinant RBD protein. A representative test is shown in Figure 1c, in which 13 known negative feline sera samples from the N-based ELISA were used to determine the cutoff value shown as a red dashed line. Among the 19 pet cats seropositive of N-specific IgG antibodies (Figure 1b), 7 samples were found to be weakly to strongly positive for the RBD-specific IgG antibodies in the RBD-based ELISA (Figure 1c), confirming that these cats have indeed been exposed to SARS-CoV-2.

To compare the performance of N- and RBD-based ELISAs for diagnostic purpose, we simultaneously employed both assays on all 25 cat sera received from June 5 to 12, 2020, along with a few N-seropositive but RBD-seronegative samples as controls. As shown in Figure 1d, out of 7 sera samples that were N-seropositive, 3 were strongly RBD-seropositive and 1 was weakly RBD-seropositive. More importantly, we did not find any RBD-seropositive but N-seronegative samples, implicating that the N-based ELISA is more sensitive to detect SARS-CoV-2 exposure in pet cats than the RBD-ELISA, perhaps partly due to the higher levels of N-specific IgG antibodies in the cat sera than the RBD-specific IgG antibodies. As such, we propose that N-based ELISA can be used as an initial (pilot) screening tool for serological evidence of SARS-CoV-2 exposure, which can be followed by the RBD-ELISA as a validation test.

### Screening feline serum samples by FIPV-based ELISA

A possible explanation for the presence of N-seropositive but RBD-seronegative cat sera is that the N-specific IgG antibodies present in the cat sera might cross-react with and therefore detect infection of some of the pet cats that have been infected by known feline coronaviruses (FCoVs) that appear to be prevalent among pet cats [43,44]. These FCoVs include but are not necessarily limited to feline enteric CoV and its pathogenic variant feline infectious peritonitis virus (FIPV). However, SARS-CoV-2 (a betacoronavirus) and FCoVs (alphacoronaviruses) are taxonomically distant and their N proteins share only 26% amino acid identity and 44% amino acid similarity. Regardless, in order to evaluate the potential seroprevalence of FCoVs in our cat sera and to determine the potential cross-reactivity of the coronavirus antibodies, we used commercial antigens from inactivated FIPV particles as outlined in the Materials and Methods section in the FCoV ELISA. Using this FCoV ELISA, we screened 116 cat serum samples, including the 19 N-seropositive sera samples, for potential antibodies that can cross react with FCoVs. As FIPV is the virulent form of the feline enteric CoV, the FCoV ELISA might detect both feline enteric CoV infections as well as FIPV infections. As shown in Figures 1e, 41 out of 116 feline sera samples (35%) are seropositive for FCoV antibodies, which is consistent with the known widespread FCoV infections in pet cats [43,44], and is at a much higher percentage than that of antibodies against the SARS-CoV-2’s N protein (7.9%). Taken together, these data suggest that the SARS-CoV-2 N-based ELISA can specifically detect N-specific IgG antibodies that are unlikely to be cross-reactive with the anti-FCoV antibodies in these cats. In support of this notion, as shown in Table 2, among 116 samples tested, the seroprevalence of SARS-CoV-2 N-specific and FCoV-specific IgG antibodies do not seem to correlate well. Four out of nineteen SARS-CoV-2 N-seropositive samples are actually FCoV-seronegative (21.1%), while about 26.8% of SARS-CoV-2 N-seronegative samples are FCoV-seropositive. In addition, the seroprevalence of SARS-CoV-2 N-specific IgG antibodies in the FCoV-seronegative samples (5.3%) is not much different from that of the total samples (7.9%). Taken altogether, our data suggest that the SARS-CoV-2 N-based ELISA can accurately detect N-specific antibodies independently of the potential seroprevalence for FCoV infections in these cats. On the other hand, 78.9% (15 out of 19) of the SARS-CoV-2 N-seropositive samples were seropositive for FCoV, which is a much higher percentage than that of the SARS-CoV-2 N-seronegative samples (26.8%). We surmise that this is partly due to the fact that there are likely many FCoV antigens with a relatively large diversity of epitopes for antibodies in the inactivated feline coronaviruses provided by the commercial source, which might include some of the highly conserved epitopes shared among different coronaviruses. In summary, our results demonstrate a high seroprevalence of FCoV antibodies in these pet cats and higher specificity of N-based ELISA than RBD-based ELISA in detecting SARS-CoV-2 exposure in pet cats.

**Table 2.** Seroprevalence of SARS-CoV-2 N and FCoV antibodies in feline sera

feline sera	total (#)	N sero-positive* (#)	N sero-negative* (#)	N sero-positive rate* (CI)
**total (#)**	239	19	220	7.9% (4.9, 12.1)
**FCoV sero-positive (#)**	41	15	26	36.6%
**FCoV sero-negative (#)**	75	4	71	5.3%
FCoV sero-positive rate* (CI)	35.3%	78.9% (54.4, 93.9)	26.8% (18.3, 36.8)	

* Proportions are reported with 95% confidence interval (CI) computed using the Clopper-Pearson method.

### Determination of SARS-CoV-2 neutralization titers in cat sera

**Figure 2.**
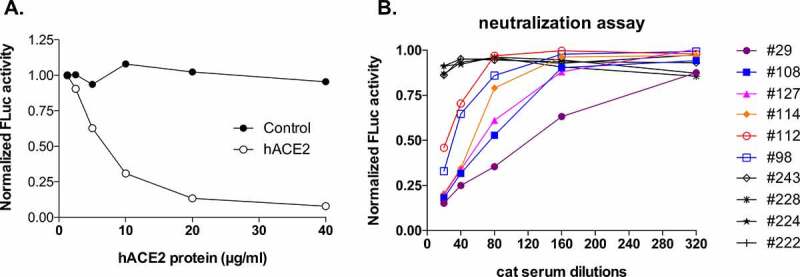
**Quantification of SARS-CoV-2 neutralizing antibodies (nAb) in pet cat sera**. The SARS-CoV neutralizing assay was conducted using a SARS-CoV-2 S pseudotyped replication-defective VSV expressing the firefly luciferase (FLUC) reporter gene. The FLUC activity was measured at 24 h post-infection and normalized to control wells (and set as 1). Each sample was measured in technical duplicates. (a) The pseudotyped virus entry is dependent on SARS-CoV-2 host receptor hACE2. The neutralization assay was conducted with increasing concentrations of recombinant hACE2. (b) A representative neutralization assay of pet cat serum samples. Two independent experiments were conducted

To determine whether neutralizing antibodies can be developed in the seropositive pet cats, we conducted a virus neutralization assay to determine whether cat sera could inhibit a recombinant VSV expressing firefly luciferase reporter gene that is pseudotyped with the SARS-CoV-2 spike (S) protein (rVSVΔG/Fluc-CoV2-S) [38] from infecting human kidney epithelial 293 T cells stably expressing hACE2. To ensure that this assay works effectively, we first incubated cells with a mixture of the reporter virus with increasing concentrations of the recombinant hACE2 protein, which is the host receptor for SARS-CoV2, and showed that there was a dose-dependent inhibition, demonstrating the specificity of this assay to evaluate SARS-CoV-2 entry (Figure 2a). Using this assay, we next evaluated the neutralizing potential of the selected seropositive cat sera, which included all 19 N-seropositive samples as well as samples representing different groups, such as N-seronegative but FCoV-seropositive, and both N- and FCoV-seronegative samples. To do this, serial dilutions of the respective sera samples were incubated with the reporter virus before adding onto the target cells. As shown in Figure 2b, individual cat serum exhibited differential potential to inhibit the reporter virus entry into cells. Some cat sera (#243, #228, #224, and #222) did not show any neutralizing activity even at the lowest dilution factor (1:20), while other cat serum samples showed various levels of neutralizing activities with the strongest one being detected for serum sample #29. We quantified the neutralization titers for 38 total cat serum samples, including all 19 SARS-CoV-2 N-seropositive samples, 6 N-seronegative but FCoV-seropositive samples, and 13 N- and FCoV-seronegative samples (Table 3). SARS-CoV-2 neutralizing activity was detected in a total of 15 of those cat sera, all of which are N-seropositive and 7 of which are RBD-seropositive. Our results suggest that besides targeting the RBD-associated neutralizing epitopes of the SARS-CoV-2 spike (S) protein, a high percentage of the neutralizing activities in cat sera also targets non-RBD region and that the RBD-based ELISA is less sensitive than the N-based ELISA to detect SARS-CoV-2 associated IgG antibodies in pet cats.

**Table 3.** ELISA results and neutralization titers of feline serum samples

(ND: not detected).

### Individual and pooled ELISA screenings of canine serum samples

In addition to feline serum samples, we received canine sera from the same veterinary medical center during the same period of times (Table 4). We initially used the SARS-CoV-2 N-based ELISA to screen the first 210 dog sera received from 24 April 2020 and identified 4 seropositive samples (1.9%). Due to the relatively low seroprevalence of the N-specific IgG antibodies in dog sera and the need for screening a larger number of pet dog samples than pet cat samples in order to ascertain the results, we decided to develop a pooled ELISA screening method, in which a pool of 5 dog serum samples was used in each test well of the 96-well plate.

**Table 4.** Canine serum samples screened by SARS-CoV-2 N- and RBD-based ELISAs as well as for SARS-CoV-2 neutralization

canine serum ID#	Collection date	N ELISA (#)	N sero-positive (#)	N sero-positive rate* (CI)	RBD sero-positive (#)	RBD sero-positive rate* (CI)	nAb titer
**1–38**	4/24/20 – 4/27/20	37	0	0.0% (0.0, 9.5)	0	0.0% (0.0, 9.5)	
**39–108**	4/28/20 – 5/3/20	71	1	1.4% (0.0, 7.6)	0	0.0% (0.0, 5.1)	ND
**109–410**	5/4/20 – 6/2/20	302	3	1.0% (0.2, 2.9)	0	0.0% (0.0, 1.2)	ND
**411–511**	6/5/20 – 6/12/20	100	1	1.0% (0.0, 5.4)	0	0.0% (0.0, 3.6)	ND
**Total**		510	5	1.0% (0.3, 2.3)	0	0.0% (0.0, 0.7)	

* Proportions are reported with 95% confidence interval (CI) computed using the Clopper-Pearson method.

(ND: not detected).

**Figure 3.**
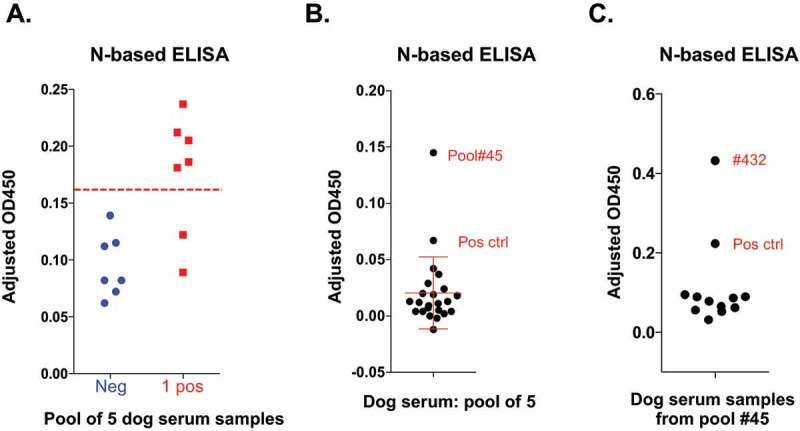
**Serological tests of pet dog sera**. (a) Validation of the pooled N-based ELISA test. A pool of 5 pet dog samples were tested in the standard dog N-based ELISA as described in Materials and Methods. The negative control (Neg) consists of all 5 N seronegative samples confirmed by previous individual ELISA test. The positive control (1 pos) consists of 4 N seronegative samples and 1 seropositive sample. The cutoff OD_450_ value is shown in a red dashed line. (b) Testing of pet dog sera by the pooled N-based ELISA. The positive control (pos ctrl) consists of one confirmed N seropositive serum and four confirmed N seronegative sera, and a seropositive pool (#45) are shown. (c) Identification of the seropositive pet dog sample in pool #45 by individual N-based ELISA. A positive control (pos ctrl) and the seropositive sample #432 are shown. Each sample was measured in technical duplicates

First, in order to determine the sensitivity and specificity of this pooled testing method, we used various known seronegative and seropositive dog’s serum samples that have been screened by the N-based ELISA described above to set up seven sets of negative controls with each pool containing five randomly selected N-seronegative pet dog’s serum samples, as well as a pool of positive control that consisted of four known N-seronegative pet dog’s serum samples and a known N-seropositive dog’s serum sample. As shown in Figure 3a, there was a statistical significance between the positive and negative pooled samples with all seven pooled negative controls showing consistently low levels of OD_450_ values, while five out of seven (5/7) pooled positive controls showing OD_450_ values that were above the cutoff OD_450_ value of 0.178, suggesting that this pooled sampling method was highly specific and had a sensitivity rate of approximately 71%.

Using this method, we conducted pooled testing of the remaining 300 dog serum samples and found that only one pool (#45) was seropositive for N-specific IgG antibodies (Figure 3b). To confirm the result and to identify the individual dog serum in this pool that is responsible for seropositivity, we conducted individual testing of all five samples in this pool #45 along with several known seronegative dog’s samples and one known dog’s seropositive sample and identified dog’s serum sample #432 as the seropositive sample in this pool of dog’s sera (Figure 3c). From both individual and pooled testing methods, we identified a total of five out of 510 (5/510) N-specific seropositive samples, which implicates a very low seropositivity rate of about 1% (Table 4). We tested 28 dog serum samples, including the 5 N-seropositive samples for RBD ELISA. It is noteworthy that none of the five N-seropositive pet dog’s serum samples were RBD-seropositive in the SARS-CoV-2 RBD-based ELISA or could exhibit any neutralizing activity in the SARS-CoV-2 spike-pseudotyped vesicular stomatitis virus (VSV)-based neutralization assay (Table 4). Collectively, our results demonstrate a relatively low level of seroprevalence of SARS-CoV-2 exposure in pet dogs and a limited neutralizing antibody response in even the few seropositive dog’s sera.

## Discussion

It has been demonstrated that multiple animal species, including dogs and cats, are susceptible to SARS-CoV-2 infection under experimental conditions or natural settings [35]. However, little is known about the prevalence of SARS-CoV-2 infection of companion animals in households. We have successfully generated various ELISAs to assess antibodies against different proteins of feline coronaviruses (FCoVs) and SARS-CoV-2, as well as a convenient SARS-CoV-2 spike-pseudotyped vesicular stomatitis virus (VSV)-based neutralization assay to evaluate the levels of neutralizing antibodies in the serum samples of pet cats and pet dogs in the state of Minnesota during the early days of the COVID-19 pandemic (mid-April to mid-June of 2020). Altogether, we analyzed the seroprevalence of SARS-CoV-2 antibodies in 239 pet cats and 510 pet dogs, of which serum samples were collected at a veterinary medical center of the University of Minnesota, Twin Cities during the time when the local human COVID-19 positive cases increased steadily but remained relatively low with 7-day average cases in MN from 2 to 37 [42]. Our results showed that the seroprevalence of SARS-CoV-2 in pet cats appeared to be higher (11%-12%) during the period between May 8^th^ and June 12^th^, 2020 than in the earlier days of the pandemic (April 16^th^ – April 27^th^, 2020), whereas the seroprevalence in pet dogs during these periods of time remained relatively low (1.0%). Our data is consistent with previous study showing that SARS-CoV-2 replicates poorly in dogs but efficiently in cats and that cats but not dogs can transmit virus through aerosols [14,17,19]. The mechanism for the differential susceptibility of dogs and cats to COVID-19 has not been well understood, but dogs are found to have low ACE2 expression in the respiratory tract [45].

Our results suggest that cats are quite susceptible to natural SARS-CoV-2 infections, consistent with several prior studies. An early study in Wuhan, China, after the COVID-19 outbreak showed 15% seroprevalence of SARS-CoV-2 RBD IgG in cats [46]. A large-scale study conducted on 919 companion animals in Italy found 3.3% of dogs and 5.8% of cats with measurable SARS-CoV-2 nAbs but no animals tested PCR positive [47]. Direct human-to-cat transmission was identified in 6 out of 50 cats from COVID-19 households or close contacts in Hong Kong [48]. A preprint study of dogs and cats living with COVID-19 patients in Texas found 47.1% of 17 cats and 15.3% of 59 dogs were positive for SARS-CoV-2 via viral RNA or nAb detection [49].

In our study, the companion animals were presented to the veterinary medical center for various medical conditions or wellness checks. At the time of admission or presentation, there was no known or perceived association of COVID-19 disease in these patients. As the animal sera used in this study were discarded and archived samples, we could neither determine the status of viral infection in animals at the time of admission by viral RNA testing nor were we aware of the owners’ health information. Retrospectively, a significant percentage of SARS-CoV-2 seropositive cats had digestive signs (vomiting, diarrhea) but rarely respiratory signs at the time of admission. However, it is less likely that these symptoms are directly caused by SARS-CoV-2 infection, because domestic cats experimentally inoculated with high dose of SARS-CoV-2 do not develop any clinical diseases [17–19], suggesting that cats are likely asymptomatic carriers of SARS-CoV-2, as has recently been reported [49,50].

It is important to note that in our study, the N-based ELISA demonstrates higher levels of sensitivity to detect SARS-CoV-2 exposures in companion animals than the RBD-based ELISA. Most SARS-CoV-2 serological tests can detect antibodies against the viral N and S protein (either partial or full-length) and show generally high levels of specificity and sensitivity against SAS-CoV-2 in human sera [51,52]. However, most if not all of these serological tests have not been carefully evaluated for use in other animal species, including companion animals. Using ELISAs that are based on the full-length RNA-free SARS-CoV-2 N and RBD proteins as well as SARS-CoV-2 nAb assay, we have been able to directly compare the levels of sensitivity and specificity between our N- and RBD-based SARS-CoV-2 ELISAs to detect anti-SARS-CoV-2 IgG antibodies in pet cats and dogs. The RBD-specific IgG ELISA appears to be highly specific, as all 7 RBD seropositive cats are also positive for N-specific IgG and nAbs (Table 3). However, at least 8 RBD-seronegative cat sera (3% of total samples) are positive for both N-specific IgG and nAbs (Table 3), suggesting that the RBD-based ELISA is not very sensitive and fails to detect at least 50% of SARS-CoV-2 exposures in pet cats. On the other hand, our in-house N-based ELISA is highly specific and sensitive to detect SARS-CoV-2 exposure in pet cats and dogs. Though we cannot determine the actual number of infected vs. non-infected cases in this study due to the lack of the longitudinal data of viral RNA detection, we, however, can deduce the level of specificity and sensitivity by cross checking the results among different screening methods, N- and RBD-based ELISA, SARS-CoV-2 nAb, and FIPV ELISA. Comparison of the N and FIPV ELISA (Tables 2 and 3) suggests that the N seropositivity is unlikely due to the cross-reactivity with FCoV antibodies. Similarly, canine CoVs were reported to be about 55% seroprevalence in North American dogs and, up to 76% and 86% in the UK and Italy, respectively [53–55]. Given the ubiquitous presence of canine CoVs, the low level of SARS-CoV-2 N seroprevalence (1.0%) in our tested dog samples is unlikely caused by cross-reactivity with canine CoVs. Furthermore, most of the N-seropositive cat sera (15 out of 19, 79%) have been corroborated by the presence of SARS-CoV-2-specific nAbs (Table 3). In the remaining N-seropositive but RBD- and nAb-negative cases (4 cat sera and 5 dog sera), they likely represent limited SARS-CoV-2 antibody responses after low levels of virus exposure in those animals. Correspondingly, human COVID-19 sera have shown variable levels of IgG antibody responses against the viral N and S antigens with distinct kinetics [56–58]. In particular, a significant percentage of the COVID-19 patients exhibit only mild symptoms (20%) and do not develop S1-specific IgG antibodies [59] or nAbs [56,57,60]. Taken altogether, our results suggest that SARS-CoV-2 N-based ELISA is a specific and more sensitive test to detect SARS-CoV-2 exposures in animals such as pet cats and dogs than the RBD-based ELISA.

A comparison of antigen-binding IgG antibodies and nAbs suggests that the level of RBD-binding IgG antibodies is not a reliable assay to evaluate the level of neutralizing capacity in companion animals. Although RBD is the main target of the SARS-CoV-2 neutralizing activity in human sera [60], other epitopes on the remaining parts of the S protein, such as S1 N-terminal domain (NTD) and S2, can also be targeted by nAbs [61]. We have shown that 50% of cat sera with positive nAbs do not have detectable RBD-specific IgG (Table 3) and that, even though all RBD-seropositive samples exhibit neutralizing capacity, their nAb titers are generally low (1:20 to 1:60), and in some instances, lower than some of the RBD-seronegative samples (1:120). Our results therefore suggest that at least 50% of SARS-CoV-2 neutralizing activity in pet cats targets non-RBD regions of the S protein and caution the exclusive use of RBD-specific IgG antibodies in the evaluation of vaccine-induced immune responses.

In summary, our study provides the first results of serological tests of pet cats and dogs in midwestern USA during the early phase of the COVID-19 pandemic in the state of Minnesota. Our results demonstrate that pet cats are more susceptible to natural SARS-CoV-2 infection than pet dogs in MN early in the COVID-19 pandemic and that SARS-CoV-2 N-based ELISA is more specific and sensitive assay to detect SARS-CoV-2 exposures in pet cats and dogs than the RBD-based ELISA. Further studies are necessary to monitor the changes of SARS-CoV-2 seroprevalence in companion animals when human COVID-19 positive rate increases and to evaluate the potential and extent of zoonosis and potential reverse zoonosis of SARS-CoV-2 between humans and companion animals.

## Data Availability

Data has been deposited in a recognized data repository (www.Figshare.com) with a digital object identifier (DOI: 10.6084/m9.figshare.13656068).
